# The Uniting of the Nations: Will Britons in the Homeland Awake and Act?

**Published:** 1917-04-14

**Authors:** Henry Burdett


### THE UNITING OF THE NATIONS.

### WILL BRITONS IN THE HOMELAND AWAKE AND ACT?

### By Sir Henry Burdett, K.C.B., K.C.V.O.

The greatest of festivals for the Christian, Easter, has come and gone since we wrote the article on the Murder of Non-Combatants. The United States of America have joined the Allies and a state of war exists between America and Germany. The Anglo-Saxon race is thus united in resisting the effort to thrust mankind back to forms of government, political creeds, and methods of conquest which ought to have disappeared for ever from the world. General Smuts forcibly declared recently that the Boer War was supplemented and compensated for by one of the wisest political settlements ever made in the history of the British Empire. That fact should prove a good augury for the future of the British nation and Empire, and I purpose to-day to try to forecast a little some of the many things which we in the Homeland, as individuals who may be kept out of active participation in the fighting, should give our minds to in preparation for the victorious termination of the war. The conclusion of the war, if we are in **deed** and in **truth** a Christian people, will mean the creation of a real, living, splendid Merrie England for all classes.

Those amongst us who have eyes to see and brains to think, and who use both continuously, must have spent some time at least on an examination of the mass of legislation which has had its being and enactment in the course of the Great War. The effects of that legislation have been to sweep aside convention, and to expose with relentless force what is realised by very few Britons, that the procedure of the House of Commons, supported by tradition and given outwardly by politicians a specious elevation resting upon sacred laws too deep-seated and too high to be mended, much less ended, has been revealed as largely make-believe. In fact they have been mainly mere barriers and machinery used by politicians at their will, to delay and hamper so-called legislation, and to render it impossible, or nearly impossible, for the progressive development of the people, through legislation, with the aid of Parliament, to be cultivated, developed, and made a living force to ensure the prompt attainment of reforms and pressing improvements. The existing conditions of life for many residents in Great Britain and Ireland causes the lives of large masses of the inhabitants to be threatened by grievous dangers to health, to life, and to much that makes life on this planet best worth having.

Politicians for decades of years have been very fond of talking, and journalists of writing on the pressing needs and wrongs of the population, on the laws of health, their shameful neglect, and much else, but to little effect. The provision of real homes, in buildings of the highest hygienic efficiency, full of facilities for the preservation of health, the enjoyment of life, the maintenance of moral laws, the cheer and brightness, without which home and family life in Christ's meaning of the words are impossible, are, alas! still largely absent almost everywhere, not only in crowded centres, but in country districts, villages, and hamlets. Add to these abominations, worthy only of a non-Christian people, the further fact that infantile mortality in these islands in many places, and generally, has been and is so much too high as to justify a charge of criminal neglect and wanton cruelty against some property owners, legislators, and local authorities from the highest to the lowest. By tolerance of such evils bishops, priests, clergy, and ministers of religion of all types, in conjunction with laymen of every rank, are largely responsible. Without going further, the call for preparation for drastic action and remedy to every individual citizen amongst us is paramount. The state of affairs existent in Great Britain to-day in these respects cannot and must not survive an avoidable hour longer than the promptest action of the people of these islands, through their spontaneous and united action, can shorten by changing all these things and making them once for all worthy of the Land of the Free, of the Mother Country of many nations of English-speaking stocks who, with India, are directly responsible for one-third of the globe, who has behind her to-day in support of her policy of the future of Liberty and Peace amongst nations, in conjunction with our Allies, the destinies of the peoples of the whole world. There are many other domestic matters affecting all classes of people in the British islands which equally press for early solution. It is matter for congratulation to know that the President of the Local Government Board, Lord Rhondda, is working at the practical solution on broad lines of one of them, the liquor question, with the knowledge that the inhygienic and indefensible fad of the few who stand for prohibition is to be swept aside, and that the public-house, the poor man's hotel, is to be made a wholesome, health-giving, pleasant, and enjoyable centre for the many, who will be able to use and enjoy it, to their comfort, well-being, and development, to an extent at least equal to that which other classes of people have been able to utilise the clubs they have created for their convenience as places of association, information, and pleasure.

We have taken these few examples of where our England to-day is wanting in completeness and Christian standards, owing to the circumstances that we as a nation have permitted large masses of the population to grow up who unaided are unable in existing conditions to have to the fullest possible extent the health-giving advantages, the wholesome surroundings, the facilities for enjoying life and freedom, the comforts of the home which are essential to enable them to live a full life in pursuance of Christ's teaching, and the upholding of the principles on which rest most things which make the doctrine of immortality desirable, and the spiritual life of the individual attainable to the full. There are many other essential changes, too numerous to mention, which the Motherland must promptly effect for the welfare of her own children if she is to justify the position in which she has been placed by Providence, through the verdict upon her policy passed by the nations of the earth and their peoples. These nations have wholeheartedly joined hands with the Motherland and British Empire in upholding, to use the words of the President of the United States, "democracy, for the right of those who submit to authority to have a voice in their own government, for the rights and liberties of small nations, for the universal dominion of right by such a concert of free peoples as will bring peace and safety to all nations and make the world itself at last free." Free England must therefore herself be freed. Every one of her children must not only be free, but be placed in a position to live his and her life in full vigour and blessedness, subject only to the conditions which will make this possible for every one of them by guarantees and enforcements.

### The Individual and the Community.

A National Mission has been proceeding amongst us for many months, with a view to awaken the consciences and minds of members of all denominations and people generally, to the call there is upon every resident in the Homeland to apprehend that upon him and her rests the responsibility of preparation, by the cultivation and recognition of their spiritual nature, that our warriors, naval and military, when they return after the war, may find those at home in tune with their own spiritual awakening, and the recognition of man's dependence upon Almighty God, His guidance and help in all the varying circumstances of human life. It is a matter for rejoicing that the Churches should themselves have spontaneously identified themselves with the recognition of the fact that the Motherland needs the active co-operation and whole-hearted, energetic, convinced support of all her sons and daughters. These, by the awakening of their intelligence and active interest in everything that affects the welfare of their neighbours and by their mastery of the underlying causes which, in the circumstances existing before the war, have suffered the evils we have indicated and others, will thus become practically acquainted with their own needs and the needs of the humblest of their neighbours. The hope is, as the crisis demands, that men and women will place themselves in a position to unite to secure that after the war the British Isles shall be indeed the land of the free, where all classes are united to secure, as the first thing before everything else in our daily life, the happiness, welfare, progressive and moral development and upbuilding of the most defenceless members of the race. It must nowhere any longer be regarded as *opéra bouffe* finance to put the claims of the worker, his rights and adequate remuneration as the first charge upon every business, enterprise, and employment which, for its success, must have the continuous output of service and labour, or both, from men and women in the production of the commodities without which the business could not succeed.

We hope we have made it clear that although the men and women as individuals, who are now resident in these islands whilst our warriors are fighting in defence of the policy we are all united to uphold, have each a very real, purposeful, and definite responsibility to bring their minds to bear on all these matters. The ultimate adjustment and promulgation of such questions must find expression through Representative Gatherings or Societies or Circles, into which the community amongst whom they reside must necessarily be organised and divided, to make it possible that the whole voice and vote of the majority shall be made effective in results, immediately, after the war. And here it is essential to point out that in the British Islands at the present time democracy has in reality no voice. Up to fifty years ago the British Parliament was free to express the will of the people, and did express it, as history records. But since that time, with two notable exceptions, the case of Home Rule and the recent crisis, the House of Commons has never expressed the will of the people and made it effective by turning out a Ministry with which the nation was dissatisfied. That is so because party and party government have superseded for practical purposes the rights and power formerly vested in the people, which they have lost largely because politics are purse-ridden in all their circumstances in the present day, and money, the root of all evil, has become the curse of politics, of patronage, as well as the oppressor of democracy and the obstructor of the free will of British men and women. There is one cheering fact in this connection, and that is the continuous development of municipalities, and the widely extended influence of sound local government and control by the best municipalities. The lighting, water supply, housing, health, education, and a multitude of other developments have in the best-managed municipalities produced some splendid results. Unfortunately, municipal government in these islands is not wholly free from politics and graft—the latter is a national danger and disgrace still, especially as affecting small house property, and the homes of the humbler citizens. To have our England the Home of the Free for All Her People, every taint of party politics and graft must be eliminated by the citizens from municipal government. Here again the immediate and present essential which every man and woman in the Homeland should take to heart and understand is, that, it is for each one of them to think out these things and prepare to make their voice and vote effective for cleansing the Homeland and her Government in the directions I have indicated. In no other way can the good and freedom of every resident in these islands be made equally secure, when the great change and opportunity arrives by the attainment of victory and the conclusion of the only peace we can accept—a peace that will destroy the present

**THE UNITING OF THE NATIONS—** *(continued).*

natural foe to liberty, and make the world safe for democracy.

What is here written is the result of a life's study of social relations and the actual conditions of life amongst all classes of the community in the British Isles. The application of the principles upon which we have ventured to lay stress means that every class, from the highest to the lowest, in fact, do to-day, as we believe, desire that the British nation shall celebrate the establishment of peace by the united action of all classes to secure that after the war England, the Land of the Free, shall in all respects be really, demonstrably, incontrovertibly free for all, in the interests of all. Our fight for freedom in this war, through our warriors on the seas and the land, through the enormous armies which we, a relatively small island people, have raised in fulfilment of the nation's determination to secure freedom for the nations, has brought us the inestimable and glorious revolution of an entire elimination of all social distinctions between man and man wherever our fighting men have found themselves associated, in fulfilment of the nation's will. Is there a man or a woman at home who is worthy of the name, or the opportunity which each has at the moment to prepare for after the war, who cannot thank God and take courage that these class distinctions have disappeared from amongst our fighting men? Is there one of them who will not, whatever the social position of each, high or low, influential or simple, be willing and ready to work continuously to provide that no class prejudice or hindrance is permitted anywhere to interfere with the progressive gathering together of individual British people in the Homeland into Circles in each community? These Circles to facilitate and organise, with the view of enabling each individual to become, through knowledge and appreciation, qualified by his and her action and vote after the war to make Great Britain the land of the free for all her children and, in God's mercy, blessed for evermore?

We look forward to a Great Election after peace is declared, and to seeing three types of candidates, (1) those who will stand on the independent choice and nominees of the people in every constituency everywhere throughout the country to represent the free and untrammelled wishes and will of the democracy, as opposed to the choice of the politicians and political parties and their machinery. In addition we suppose in every constituency there will be political candidates of at least two types, representing political parties, organisations, etc. But, if the people are wise in this Great Election of Re-Birth and Purification. most candidates, not chosen independently by the people, may become conspicuous in history owing to the smallness of the votes which party funds and party organisation were able to muster at the polls for them on the great day. The choice of the representatives of the free citizens of all classes in each constituency should be made by taking the men and women who have done the public work in that centre of population for the community in local and parliamentary affairs, to the benefit of the community, and have shown, as the late Mr. Joseph Chamberlain and others have shown, marked ability in administration and representation, with a will to anticipate and give fruitful expression to the wishes of the people generally amongst whom they have lived and worked.

It has been said to us that already what we here urge has been hazily said and talked about generally throughout the country. Will the reader please realise that the time for talk is over, and the hour for decision and action has come? Everything must have a small beginning, but if every man or woman who reads what is here written will really awake to his or her responsibility, and to the fact that the hour for action is about to strike, they will forthwith exert themselves to form, first a small organisation of a few to begin with, next they and their friends will make it their business to gather together other groups of friends, and then form a Circle for the Centre or District where they reside. If these steps are taken it should be relatively easy to settle steadily down to work out the plan here formulated. Then the Motherland will have cause to be proud of every one of their pioneers. For their action should make every individual Briton join the Pioneers, and thank God and take courage for the opportunity, the education, and the splendid fruit which may result from their awakenment and action to England and the Empire.

